# A Systematic Review Examining the Association of Falls With Diabetes‐Related Foot Ulcers

**DOI:** 10.1002/jfa2.70057

**Published:** 2025-06-16

**Authors:** Mike Wu, Mallika Sinha, Chanika Alahakoon, Kristen S. Barratt, Shivshankar Thanigaimani, Jonathan Golledge

**Affiliations:** ^1^ Queensland Research Centre for Peripheral Vascular Disease College of Medicine and Dentistry James Cook University Townsville Australia; ^2^ The Department of Vascular and Endovascular Surgery Townsville University Hospital Townsville Australia; ^3^ College of Medicine and Dentistry James Cook University Townsville Australia; ^4^ College of Medicine and Dentistry James Cook University Cairns Australia; ^5^ The Australian Institute of Tropical Health and Medicine James Cook University Townsville Australia

**Keywords:** diabetes, falls, neuropathy, ulcer

## Abstract

**Background:**

The aim of this study was to systematically review the risk of falls in people with diabetes‐related foot ulcers (DFU).

**Methods:**

A systematic search of Medline, Pubmed, Embase, Cochrane and CINAHL was undertaken to identify observational studies reporting falls and containing a group of people with a DFU and a control group with diabetes but no DFU. Risk of bias was assessed by a modified Newcastle–Ottawa Scale. Meta‐analysis was performed using a random effects model.

**Results:**

Four studies involving 3643 participants with a DFU and 42,436 participants with diabetes but no DFU were included. A meta‐analysis showed high heterogeneity between studies (*I* ^2^ = 95%) and an increased risk of falls in people with DFU (risk ratio 2.25 and 95% CI 1.05–4.84). One study had a low risk of bias and three studies had a high risk of bias. Leave‐one‐out analyses showed that exclusion of the study with the largest effect on heterogeneity resulted in a risk ratio of 1.80 (95% CI 1.33–2.43 and *I* ^2^ = 0%).

**Conclusions:**

Currently available evidence suggests people with a DFU have a higher risk of falls but most past studies have a high risk of bias. Further well‐designed cohort studies are required.

## 1 Introduction

Accidental falls become more common as people age [1]. Up to one‐third of older people living in the community fall at least once each year [2]. Falling leads to a reduction in quality of life due to fear of further falling [3] and loss of independence, which is often due to self‐imposed restrictions to avoid falling [4]. Falls can also lead to injury, fractures, hospital admission and death [5].

A population in which accidental falls may be under‐appreciated are those with diabetes. Older adults with diabetes have a high risk of falls, reported to be 60% higher than healthy controls in some studies [6, 7]. This likely results from gait and balance dysfunction [8], visual impairment [9, 10], cognitive decline [11, 12] and the complications of prescribed medications [13, 14].

In diabetes, the mechanisms that cause falls are well‐understood. Gait and postural control are impaired due to the loss of ankle proprioceptive feedback from diabetes‐related peripheral neuropathy [15, 16]. There is greater unsteadiness due to vestibular dysfunction [17]. Diabetes causes the accelerated onset of cognitive decline, which hampers the ability to navigate a complicated external environment [18] and leads to slower reaction times [19]. The ability to perform corrective movements to prevent a fall is impaired by sarcopenia [20, 21] and proximal lower limb weakness [22]. These factors have a complex interplay, which is reflected by the absence of an accurate predictive model to assess falls risk in patients with diabetes [23].

People with diabetes‐related foot ulcers are potentially a higher‐risk population for falls due to their frailty and the need for offloading treatment of the ulcer. Diabetes‐related foot disease is associated with poorer physical function [24, 25]. Also, restriction of mobility occurs during offloading treatment of foot ulcers, promoting physical deconditioning and increasing the likelihood of discharge to a higher level of care due to mobility concerns. Also, this population is commonly treated with specialised offloading devices, such as irremovable knee‐high offloading devices, insoles or rocker‐bottom shoes, which may pre‐dispose to falls [26].

Understanding the falls risk of the diabetes‐related ulcer population is important because it may alter the way we manage these patients. Firstly, many people with diabetes‐related foot ulcers have concomitant artery disease that requires treatment with lifelong blood‐thinning medication, meaning falls can have devastating consequences such as intra‐cranial bleeding [27]. Secondly, falls leads to fear of falling and self‐imposed mobility restriction [4]. After an ulcer heals, patients spend less time on their limb, resulting in a reduced quality of life and increased risk of frailty.

Prior research has assessed the risk of falls conferred by diabetes‐related foot ulcers using surrogate measures. Balance or falls risk scores, such as the Turkish Morse Fall Scale [28], Downton Index [29] and Tinetti Falls Efficacy Scale [30], have been used, but there are few studies that have directly examined the falls incidence. Furthermore, there has been no prior systematic review of falls risk in people with diabetes‐related foot disease. This systematic review aimed to assess the risk of falls in people with diabetes‐related foot ulcers as compared to people with diabetes but no foot ulcer.

## 2 Methods

### 2.1 Protocol and Focus

The protocol for this systematic review was created on 12/3/2024 prior to commencing data collection and registered on PROSPERO as CRD42024525604. This systematic review was reported according to the PRISMA 2020 statement [31].

### 2.2 Criteria for Inclusion

Observational studies and retrospective studies were included. Interventional studies and reviews were excluded. Studies authored after the year 2000 were included to better reflect contemporary disease and treatment. There was no language restriction. Reports were considered if their full text was available, and unpublished papers and conference abstracts were also included.

People aged 18 or over with diabetes identified via confirmatory laboratory tests, anti‐hyperglycaemic medication use or a reported history were included [32]. The exposure group consisted of anyone with active diabetes‐related foot ulcers. The control group could consist of patients with prior or no history of diabetes‐related foot ulcers who had no current ulcer. Patients recruited from both inpatient or outpatient settings were considered, and patients with a wide‐ranging ability to manage their personal daily activities were considered. Patients with a history of major lower limb amputation were excluded. Reports that collected information on falls incidence were included.

### 2.3 Outcomes

Falls defined as an unexpected event where an individual comes to rest on the ground, floor or a lower level without a known loss of consciousness was the primary outcome [33]. This could have been reported as the number of patients falling or the number of falls in each group to produce a frequency comparison. The secondary outcomes were falls‐related complications, such as falls‐related hospital admission and fractures.

### 2.4 Search Strategy

To identify eligible studies, a literature search was conducted between March and July 2024 and the date range for papers was limited to 20.0–2023. Medline, Pubmed, Embase, Cochrane and CINAHL Complete were searched. In consultation with a librarian, highly inclusive search strategies were created for each database to increase the likelihood of finding all relevant articles, accepting that there would be more manual screening required. The following MeSH terms and key words were used: (‘diabetes complications’ OR ‘diabetic foot’ OR ‘diabetic neuropathies’ OR ‘foot ulcer’ OR ‘foot diseases’ OR ‘foot orthoses’ OR ‘diabetes‐related complication’ OR ‘diabetes‐related foot disease’ OR ‘diabetic foot ulcer’ OR ‘diabetic polyneuropathy’ OR ‘diabetes‐related autonomic neuropathy’ OR ‘diabetic neuropathy’) AND (‘accidental falls’ OR ‘mechanical fall’ OR ‘fall’). The full search string for each database can be found in Supporting Information S1: Appendix 1.

### 2.5 Screening

Endnote 21 was used to store and manage records. There was automatic de‐duplication of records via Endnote 21's record matching function, and manual de‐duplication was performed afterwards. MW and MS performed content‐based screening of the titles and the abstracts to check for articles with focus areas clearly not related to diabetes‐related foot ulcers or falls. The other articles proceeded to full text request and review. Articles were not excluded based on the study type until after the full text review, as it allowed for identification of potentially useful articles from reference lists. Two reviewers (MW and MS) independently reviewed each of the full texts against the inclusion criteria. Where it was unclear if studies were eligible, authors were contacted for further information. MW and MS recorded their decisions for each paper as ‘include’ or ‘exclude’ with a level of certainty–‘certain’ or ‘possible’, resulting in four possible outcomes for selection with reasons recorded. These decisions were made independently and any differences in the reviewers' decisions were either resolved immediately, postponed until contact had been made with the corresponding authors to request more information or referred to a third reviewer CA for arbitration.

### 2.6 Information From Other Sources

During full‐text screening, the reference lists of review articles were used to source additional studies, which were then subject to the screening. If it was thought that authors had information that could contribute to this review, they were contacted with a standardised email or message on ResearchGate. Record was made of the dates, avenue of contact and number of attempts made. Two attempts were made for each author who did not respond.

### 2.7 Data Extraction

Two reviewers (MW and KB) independently performed the data extraction using a standardised spreadsheet template. Data were collected on falls as number of events per group or number of participants at risk per group. Risk factor data were collected on the characteristics of participants (age, gender, residential status, cardiovascular risk factors, smoking status, use of mobility aids, and use of diabetes medication), disease characteristics (duration of diabetes, serum Hba1c or glucose levels, and evidence of end‐organ complications), presence of related diseases (peripheral neuropathy, peripheral artery disease [PAD]), the exposure and control groups, the study type and funding sources. Contact was made with the corresponding authors about any information that needed clarification.

### 2.8 Assessing Risk of Bias

Risk of bias was independently assessed by two reviewers (MW and CA) using the Newcastle–Ottawa Score (NOS) [34]. Normally scored out of nine, it was adapted to award an extra point to studies that were able to define a control group that had never had a prior diabetes‐related foot ulcer. The classification cutoff points were left unchanged, and this allowed studies to achieve a classification of a lower level of bias more easily than the original scoring system.

There were 10 criteria in our modified NOS (Supporting Information S2: Appendix 2), split between the three domains of selection, comparability and outcome. Studies scoring 7–10 were classified as low risk, 4–6 were high risk and 1–3 were very high risk. MW and CA discussed any differences in their scoring for each article, and a consensus score was achieved with some arbitration from a third reviewer JG.

### 2.9 Synthesis

To be included in the meta‐analysis, studies needed to report the primary outcome as the number of fallers or the number of falls in each group. The studies needed to have a control group of participants with diabetes and a group of interest with diabetes‐related foot ulcers. Meta‐analysis was performed using the inverse variance method with random effects model, and the Hartung–Knapp method was used to calculate the confidence intervals (CIs) due to the small number of available studies. The primary outcome was the difference in falls between each group. This was calculated and reported as risk ratio (RR) with 95% CI and presented as a forest plot. Statistical heterogeneity between studies was assessed using the *I* ^2^ statistic and interpreted as low (0%–49%), moderate (50%–74%) or high (75%–100%) [35]. Sensitivity analyses via the leave‐one‐out analysis approach were performed to test the heterogeneity of the included studies and the consistency of the findings. Meta‐analysis was conducted using the ‘meta’ package and sensitivity analysis was conducted using ‘dmetar’ package of the R software version 3.4.4. A funnel plot was used to check for the publication bias.

## 3 Results

### 3.1 Search Results

**FIGURE 1:**
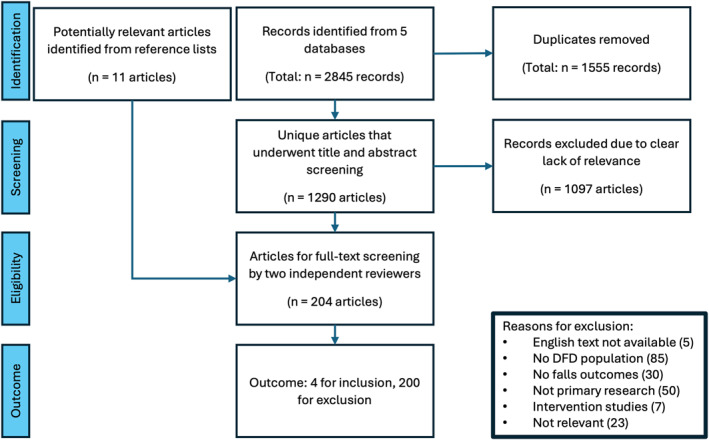
Preferred reporting items for systematic reviews and meta‐analyses flowchart.

From a total of 2845 nonunique citations found, 1290 citations remained after removal of duplicates. Following screening, 193 citations were selected for full‐text review, and the reference lists of the identified articles contributed a further 11 citations. Of the total 204 citations reviewed, 3 studies were initially included [36, 37, 38]. Contact was made to the corresponding authors of 15 studies [28, 30, 39, 40, 41, 42, 43, 44, 45, 46, 47, 48, 49, 50, 51]. Replies were received from 6 [28, 40, 42, 47, 49, 50], and the information provided by one author resulted in the inclusion of one additional study [28]. The remaining 200 studies were excluded for no English text available (5), not having a population with diabetes‐related foot ulcers (85), no falls outcomes reported (30), not being primary research papers (50), being intervention‐based research (7) or being irrelevant (23) (Figure 1).

### 3.2 Characteristics of Included Studies and Participants

All studies were published between 2017 and 2023 (see Table 1). The 4 included studies [28, 36, 37, 38] involved 46,079 patients with diabetes who either had diabetes‐related foot ulcers or did not. The sample sizes for the four studies were 44,562, 1147, 300 and 70. Two studies were retrospective cohorts with observation period of 22 and 6 years [37, 38]. The other two were cross‐sectional studies each with an outcome observation period of 1 year [28, 36].

**TABLE 1:** Participant characteristics in studies reporting falls events segregated according to diabetes‐related foot ulcers or control groups.

Reference	Group (*n*)	Age (mean ± SD)	Males	Falls history	CVD	CKD	PAD	Retinopathy	Neuropathy
Allen et al. [38]	DFU (3238)	63.5 ± 10.6	3476 (96.9)	NR	30 (0.8)	19 (0.5)	1415 (39.5)	86 (2.4)	833 (23.2)
	No DFU (41,324)	64.4 ± 11.5	39,197 (95.8)		245 (0.6)	111 (0.3)	3784 (9.2)	232 (0.6)	2489 (6.1)
Bicer [28]	DFU (83)	< 65: 42 (50.6)^e^	42 (50.6)	NR	NR	NR	69 (83.1)	NR	64 (77.1)
		≥ 65: 41 (49.4)^e^							
	No DFU (217)	< 65: 133 (61.3)^e^	82 (37.8)^d^				209 (96.3)^λ^		123 (56.7)^d^
		≥ 65: 38 (38.7)^e^							
Fang et al. [37]	DFD^a^ (262)	64.0 ± 5.2	179 (54.9)	Never	85 (26.1)	100 (30.7)	13 (4.0)	89 (27.3)	NR
	No DFD^a^ (885)	63.2 ± 5.7	579 (52.5)	Never	226 (20.5)	258 (23.4)	56 (5.1)	155 (14.1)	
Seo et al. [36]^c^	DFU^b^ (60)	64.9 ± 10.7	50 (71.4)	NR	NR	NR	49 (70.0)	33 (47.1)	46 (65.7)
	No DFU^b^ (10)								

*Note:* Data are presented as *n* (%) unless stated otherwise.

Abbreviations: CKD, chronic kidney disease; CVD, cardiovascular disease; DFD, Diabetes‐related foot disease; DFU, Diabetes‐related foot ulcer; NR, not reported; PAD, peripheral artery disease.

^a^ DFD was defined as a composite of foot ulceration, cellulitis, gangrene and osteomyelitis or paronychia.

^b^ DFD was defined as including open wounds, bleeding disorders and ulcers in the foot.

^c^ Seo et al. did not report on the baseline characteristics of their DFU and no DFU groups separately.

^d^ *P* < 0.05. *p* values were only available from Bicer et al., upon request. The other studies did not perform statistical tests for differences in the group characteristics.

^e^ This was presented as a number and percentage by the paper.

There were 3643 patients with diabetes‐related foot ulcers and 42,436 controls. 3 studies [28, 36, 38] sampled from a hospital‐based population, whereas the other study [37] was taken from a community sample. The two larger studies found their patients through ICD‐9 and ICD‐10 diagnosis codes [37, 38], whereas the two smaller studies used face‐to‐face questions and examination [28, 36].

Patient characteristic differences between DFU and non‐DFU groups for each study are summarised in Table 1. They could not be determined in Seo et al.’s study because this information was not presented. For the other three studies, the DFU and non‐DFU groups were mostly well‐matched in age and gender. Bicer et al.’s DFU group was older (49.4% above 65 years of age vs. 38.7% and *p* = 0.116) and had more males (50.6% vs. 37.8% and *p* = 0.04) [28]. Insulin use was higher in the DFU group than the non‐DFU group (83.1% vs. 62.2% in one study (*p* = 0.001) [28], 30.4% versus. 18.2% in another [37]) and diabetes duration was longer in the DFU group (58.9% had diabetes for 9 years or more compared with 44.5% [37]). For all three studies, the percentage of retinopathy and neuropathy was higher in the DFU group. Allen et al.’s study had a large difference in proportion of patients with PAD (39.5% in the DFU group vs. 9.2% in the non‐DFU group) [38]. Besides Fang et al.’s study, which excluded participants who had a prior history of falls, the other studies did not report history of falls. There was no information reported on history of minor amputations. One study showed that the DFU group used assistive mobility devices more than the non‐DFU group (54.2% vs. 23.0% and *p* < 0.001) [28]. Some studies reported on ethnicity and their groups were well‐matched in this regard. A complete summary of the patient characteristics can be found in Supporting Information S4: Appendix 4.

### 3.3 Risk of Bias of Included Studies

**FIGURE 2:**
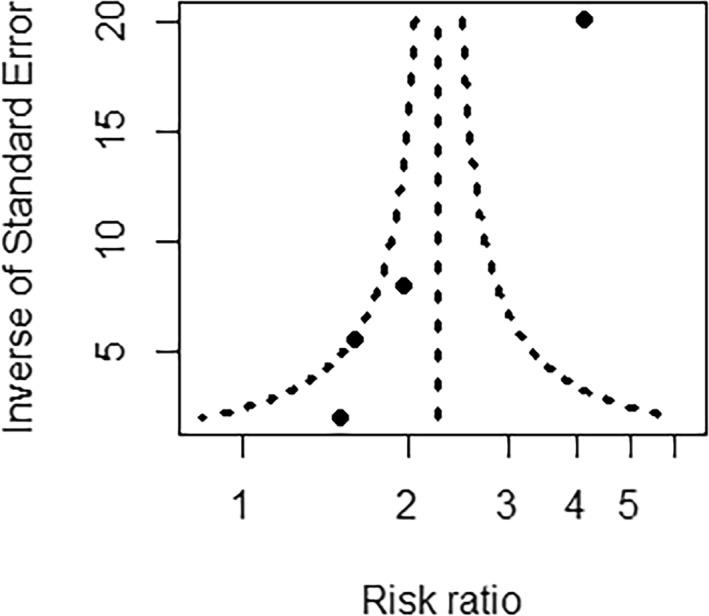
Funnel plot created using the 4 included studies.

Using a modified version of the Newcastle–Ottawa Scale, one study was deemed to be at a low risk of bias [37] and three at a high risk of bias [28, 36, 38]. All included studies performed well in the ‘selection’ domain, which had a maximum possible score total of 5, and two studies [28, 37] had information about the DFU history of its control group. Performance in the ‘comparability’ domain which had a maximum possible score of 2 was poor, with only one study using statistical models to adjust for all factors considered important for falls (prior falls, age, insulin use and peripheral neuropathy) [37] and three studies [28, 36, 38] failing to adjust for the most important risk factor (prior falls) [8]. For the ‘outcomes’ domain, the studies all scored 2 of a maximum possible score of 3. Two studies [37, 38] determined the outcome by record linkage and allowed sufficient follow‐up time for the outcome to occur but did not report on the loss to follow‐up or mean follow‐up duration. The other two studies [28, 36] had complete follow‐up due to being cross‐sectional studies but falls outcome ascertainment was unreliable due to reliance on participant self‐reporting. Detailed scoring of each study is shown in Supporting Information S3: Appendix 3. Publication bias was assessed with a funnel plot and is presented in Figure 2. There are too few studies to draw any conclusions about the publication bias.

### 3.4 The Association Between Diabetes‐Related Foot Ulcers and Falls–Individual Studies and Meta‐Analysis

**FIGURE 3:**
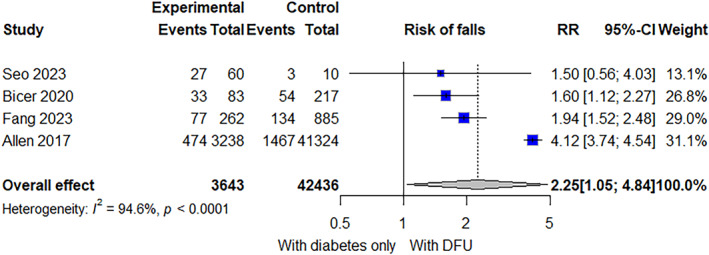
Forest plot comparing falls events in participants with diabetes‐related foot ulcers and diabetes alone.

From Allen et al.’s study [38], the risk ratio of being a faller in the DFU group compared to the diabetes group was 4.12 (95% CI 3.74–4.54). Bicer et al. [28] provided data that allowed us to calculate a risk ratio of 1.60 (95% CI 1.12–2.27). Fang et al. [37] had a risk ratio of 1.94 (95% CI 1.52–2.48). From Seo et al.’s report [36], we calculated the risk ratio of being a faller to be 1.50 (95% CI 0.56–4.03). This information is presented in Figure 3, and Table 2 has a list of the confounders that each study adjusted for shown.

**TABLE 2:** Results of the individual studies for the primary and secondary outcomes.

Reference	Group (*n*)	Confounders adjusted for	Fallers		Fall events		Falls needing hospitalisation		Falls events with fractures	
Allen et al. [38]	DFU (3238)	Age, race, gender, marital status and comorbidities	474 (14.6)	OR 2.26 (1.96–2.60)	NR		NR		NR	
	No DFU (41,324)		1467 (3.5)							
Bicer [28]	DFU (83)	Nil	33 (39.8)	OR 1.99^a^	NR		NR		NR	
	No DFU (217)		54 (24.9)							
Fang et al. [37]	DFD (262)	Age, sex, race, smoking, BMI, HTN, CKD, HDL cholesterol, triglycerides, LDL cholesterol, CVD, PAD, glycaemic control, diabetes duration, SES status and health insurance.	NR		77 (23.6)^a^	HR 1.74 (1.02–2.98)	74 (22.7)^a^	HR 1.78 (1.02–3.11)	32 (9.8)^a^	HR 1.72 (0.66–4.49)
	No DFD (885)				134 (12.2)^a^		129 (11.7)^a^		39 (3.5)^a^	
Seo et al. [36]	DFU (60)	Nil	27 (45)^a^	OR 1.91^a^	NR		NR		NR	
	No DFU (10)		3 (30)^a^							

*Note:* Data are presented as *n* (%) unless stated otherwise. Odds ratios and hazard ratios are presented with 95% confidence intervals.

Abbreviations: DFD, diabetes‐related foot disease; DFU, diabetes‐related foot ulcer; HR, hazards ratio; NR, not reported; OR, odds ratio.

^a^ Calculated by author.

**FIGURE 4:**
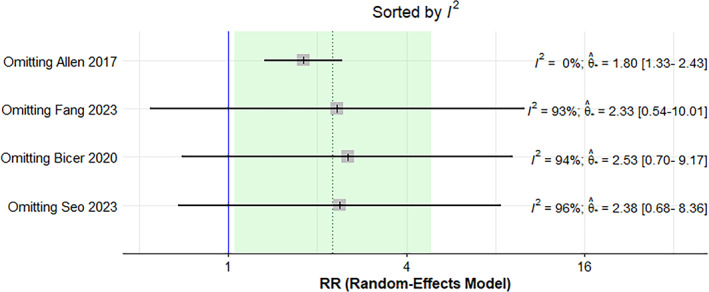
Leave‐one‐out analysis to examine the effect of each study on heterogeneity.

A meta‐analysis was performed using all four included studies consisting of 3643 patients with diabetes‐related foot ulcers and 42,436 controls. Diabetes‐related foot ulcers increase the risk of falls and there was a high degree of heterogeneity between studies (RR 2.25; 95% CI 1.05–4.84 and *I* ^2^ = 95%) (Figure 3). Leave‐one‐out analyses demonstrated that the effect size towards a higher risk ratio was influenced by one study [38] (Figure 4). The exclusion of this study resulted in a RR of 1.80 (95% CI 1.33–2.43 and *I* ^2^ = 0%).

### 3.5 Association Between Diabetes‐Related Foot Ulcers and Falls Complications

Only one study contributed results towards the secondary outcomes [37]. Fang et al. distinguished the severity of the fall, with hazards ratio for all falls, falls needing hospital admission or falls resulting in fractures being 1.74 (95% CI 1.02–2.98), 1.78 (95% CI 1.02–3.11) and 1.72 (95% CI 0.66–4.49), respectively.

## 4 Discussion

This systematic review aimed to address whether diabetes‐related foot ulcers increase the risk of falls. One of the main challenges was the scarcity of research that focused on the association between falls and ulcers. We found that studies comparing populations with and without diabetes‐related foot ulcers usually do not report falls outcomes, and studies that examine falls outcomes usually did not report explicitly on the diabetes‐related foot ulcer status of their participants. On close inspection of the protocols of relevant research papers, some studies had a data collection instrument, such as the Michigan Neuropathy Screening Instrument, that captured information about ulcer status via interview question and/or clinical examination [52]. We made contact with these authors but were either met with no response [30, 39, 41, 43, 44, 45, 46, 48, 50, 51] or were unable to analyse and present the results in a way required by this review [40, 42, 47, 49].

**FIGURE 5:**
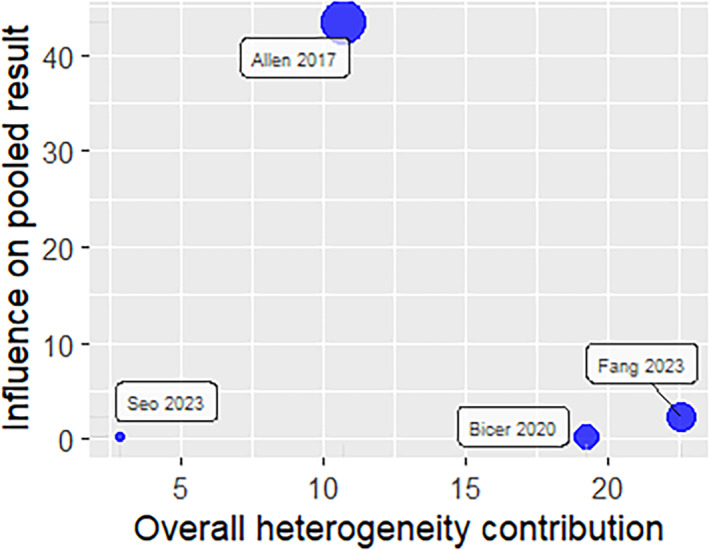
Influence of each study on pooled result and heterogeneity.

This meta‐analysis found that diabetes‐related foot ulcers increase the risk of falls, but it is difficult to know by how much as reflected by the wide confidence interval. Allen et al.’s study [38] had the largest effect size on the forest plot (Figure 3) and exerted the greatest influence on the pooled result due to the large number of study participants (Figure 5). Exclusion of this study led to a decrease in heterogeneity to *I* ^2^ = 0% and a more precise CI. Further high‐quality studies are thus needed to definitely answer the main question of this systematic review. Allen et al.’s study had a much higher prevalence of PAD in the DFU group compared to the diabetes group (39.5% vs. 9.2%). Participants with PAD are more likely to fall because PAD is associated with gait disturbance and frailty [53]. Treatment of PAD‐related ischaemic ulcers can leading to prolonged hospital admission, which can result in physical deconditioning.

Participants who undergo amputation are at a much higher risk of falls [54] and most of the studies did not exclude amputees. The lack of this exclusion criteria likely affected most of the included studies. The studies reported by Allen et al. [38], Bicer et al. [28] and Seo et al. [36] did not exclude patients with an amputation history. The study reported by Fang et al. [37] may be least affected by this confounding, because it had an incident foot ulcer group which would be expected to have a lower risk of prior amputation. It would be useful for future studies to classify the severity of PAD (e.g., using the Fontaine classification [55]), so this can be considered when interpretating the results.

### 4.1 Limitations

This systematic review included a low number of articles. Before we commenced the review, we performed a scoping review using subject headings and found few results relevant to our search. Nonetheless, we proceeded with the systematic review and improved our yield by expanding our search strategy to include ‘falls’ as a keyword and contacting authors who we thought might have collected the relevant data based on their study protocols. We restricted our search to articles published after 2000 because we thought it would be unlikely for authors from older studies to respond or still have the original data. There is a small risk that some studies were missed due to the year restriction, but given that the included studies did not reference other studies that have addressed the question previously and a recent Google Scholar search for articles published between 1950 and 1999 did not find any relevant articles, we are reassured that this risk is low.

Due to the scarcity of studies, we could not investigate the secondary outcome like we originally intended to in our PROSPERO‐registered protocol. Three studies examined falls risk scores [28, 29, 47], but they all used different numerical scales that could not be synthesised. Only one paper addressed the secondary outcome by distinguishing between minor and major falls with complications [37].

The case definition of diabetes‐related foot ulcers was felt to be reliable, as two studies [28, 36] involved face‐to‐face assessment of the participants and the other two studies [37, 38] used International Classification of Disease (ICD) codes collected for health administration purposes to identify their cases. Two studies did not have a clearly defined control group. The study by Fang et al. [37] had a clearly defined control group as they compared participants with an incident diabetes‐related foot ulcer which those who had no prior ulcer. Bicer et al. [28] reported the proportion of participants who had previously had an ulcer. However, this was not reported in by Allen et al. [38] or Seo et al. [36], resulting in the selection bias.

The way that falls were recorded varied greatly. Wallace et al. conducted a study in 2002 [56] using falls diaries and pro‐active phone calls and their incidence of falls was much higher than within the studies included in this review at 1.25 falls per person‐year. Their study was not included in this review due to the lack of a control group. The falls incidence reported by Fang et al. [37] was much lower at 16.1 (95% CI 9.9–26.3) per 1000 person‐years for the foot ulcer group because they identified falls through ICD‐9/10 codes which would not capture the minor falls that did not require a presentation to medical facility. Two studies relied on participants' self‐reporting falls [28, 36], which is subject to the recall bias. Future research can be improved by capturing falls through prospective questionnaires.

Among the older population, the major risk factors that consistently contribute to falls include a history of previous falls, impaired balance and gait and polypharmacy [8, 57]. For people with diabetes, there are the additional risk factors of peripheral neuropathy and comorbid conditions [58]. We considered a history of previous falls to be the most important confounding factor that studies needed to adjust for and gave additional credit to articles that controlled for some other factor such as age, insulin use or peripheral neuropathy. Overall, the included studies had a high risk of bias for failing to adjust for these confounders. Fang et al. [37] reported the only study that adjusted for prior falls by ensuring none of their participants had a prior history. Two studies had collected information about gait and balance disturbances and peripheral neuropathy [28, 36] but did not adjust for them. It is known that diabetes predisposes to falls via several mechanisms whereas the specific domains that need to be controlled for and their respective contribution is still an emerging area of research. Vestibular nerve dysfunction [59], postural instability [45], vibration perception threshold [60] and loss of protective foot sensation [30] all seem to be risk factors for falls in the diabetes population. It seems that it is necessary for future studies to collect a large amount of information to adjust for these known risk factors.

Some assumptions and allowances were made during the data synthesis. Firstly, in the study reported by Fang et al. [37], participants with incident diabetes‐related foot disease were compared with patients with diabetes. Most of these participants (59.2%) had diabetes‐related foot ulcers, but there were some participants with other pathologies such as foot infection and gangrene. We were unable to find out from the corresponding author the falls events in the diabetes‐related foot ulcer population only, so we used the numbers from the diabetes‐related foot disease group for our analysis. Secondly, Fang's study originally recruited 1428 participants, of which 326 had incident diabetes‐related foot disease. For their analysis on falls, they excluded those with prior falls to obtain a sample size of 1147 but did not report the size of the DFD group. We used 1147 as a proportion of 1428 to calculate the group sizes. Lastly, this study reported the number of falls events, whereas the other three studies reported the number of participants who fell. Due to the small number of studies available, the study reported by Fang et al. was not excluded from the meta‐analysis.

## 5 Conclusion

The limited literature that is available suggests that patients with diabetes‐related foot ulcers have an increased risk of falls compared to patients with diabetes alone. There is a lack of studies, which directly address this question, and the studies that already exist differ greatly in design, power and results. The increased risk of falls may depend on the foot ulcer history and PAD severity. There is a need for future well‐designed studies.

## Author Contributions

**Mike Wu:** data curation, formal analysis, investigation, methodology, writing – original draft, writing – review and editing. **Mallika Sinha:** data curation, investigation, methodology, writing – review and editing. **Chanika Alahakoon:** data curation, investigation, methodology, writing – review and editing. **Kristen S. Barratt:** data curation, investigation, methodology, writing – review and editing. **Shivshankar Thanigaimani:** investigation, methodology, formal analysis, writing – review and editing. **Jonathan Golledge:** conceptualization, investigation, methodology, supervision, funding acquisition, writing – original draft, writing – review and editing.

## Conflicts of Interest

The authors declare no conflicts of interest.

## Supporting information

Supporting Information S1

Supporting Information S2

Supporting Information S3

Supporting Information S4

## Data Availability

The full endnote library at each stage of selection and the inclusion/exclusion decisions made can be made available from the corresponding author upon request.
