# Supplementary material for: A Systematic Review Examining the Association of Falls With Diabetes‐Related Foot Ulcers
Source: J Foot Ankle Res. 2025 Jun 16;18(2):e70057. doi: 10.1002/jfa2.70057 (PMC12170942; doi:10.1002/jfa2.70057)
Supplement: Supplementary file 1 — Supporting Information S1 [file JFA2-18-e70057-s003.docx]

Appendix 1. Search strategies

Medline

Last accessed 30 March 2024

*(((MH "Diabetes Complications+") OR (MH "Diabetic Foot") OR (MH "Diabetic Neuropathies+") OR (MH "Foot Ulcer+") OR (MH "Foot Diseases+") OR (MH "Foot*

*Orthoses")) AND (MH "Accidental Falls"))*

*OR*

*((AB diabetes-related complication* OR AB diabetic foot OR AB diabetes-related foot*

*disease OR AB diabetic foot ulcer OR AB foot ulcer OR AB heel ulcer OR AB diabetic*

*polyneuropathy OR AB diabetes-related autonomic neuropathy OR AB diabetic neuropathy) AND (TX mechanical fall* OR TX fall* OR TX accidental fall*))*

*Limiters - Publication Date: 20000101-20231231; English Language; Human*

*Expanders - Apply related words; Apply equivalent subjects*

*Search modes - Find all my search terms*

*Results: 1,028*

Embase

Last accessed 30 March 2024

*('diabetic foot'/exp OR 'diabetic foot' OR 'diabetic complication'/exp OR 'diabetic*

*complication' OR 'diabetic foot ulcer'/exp OR 'diabetic foot ulcer' OR 'diabetic*

*neuropathy'/exp OR 'diabetic neuropathy' OR 'foot ulcer'/exp OR 'foot ulcer' OR 'heel ulcer'/exp OR 'heel ulcer' OR 'foot disease'/exp OR 'foot disease') AND ('falling'/exp OR 'falling') AND [english]/lim AND [humans]/lim AND [2000-2023]/py*

*Results: 1,287*

Pubmed

Last accessed 24 July 2024

(("diabetic foot"[Title/Abstract] OR "diabetes related complication*"[Title/Abstract] OR "diabetes-related foot disease"[Title/Abstract] OR "diabetic polyneuropathy"[Title/Abstract] OR ("diabetes-related"[All Fields] AND "autonomic neuropathy"[Title/Abstract]) OR "diabetic complication*"[Title/Abstract]) AND ("mechanical fall*"[All Fields] OR "accidental fall*"[All Fields] OR "fall*"[All Fields])) AND ((2000/1/1:2023/12/31[pdat]) AND (english[Filter]))

Results: 199

CINAHL

Last accessed 30 March 2024

*(((MH "Diabetes Complications+") OR (MH "Diabetic Foot") OR (MH "Diabetic Neuropathies+") OR (MH "Foot Ulcer+") OR (MH "Foot Diseases+") OR (MH "Foot*

*Orthoses")) AND (MH "Accidental Falls"))*

*OR*

*((AB diabetes-related complication* OR AB diabetic foot OR AB diabetes-related foot*

*disease OR AB diabetic foot ulcer OR AB foot ulcer OR AB heel ulcer OR AB diabetic*

*polyneuropathy OR AB diabetes-related autonomic neuropathy OR AB diabetic neuropathy) AND (TX mechanical fall* OR TX fall* OR TX accidental fall*))*

*Limiters - Publication Date: 20000101-20231231; English Language; Human*

*Expanders - Apply related words; Apply equivalent subjects*

*Search modes - Find all my search terms*

*Results: 251*

Cochrane

Last accessed 24 July 2024

*(“diabetic ulcer*” OR “foot ulcer*” OR “heel ulcer*” OR "diabetes-related complications" OR "diabetic polyneuropathy" OR "diabetes-related foot disease" OR "diabetic foot disease" OR "diabetic autonomic neuropathy" [limit to title, abstract or keywords]) AND “fall*”*

*with Cochrane Library publication date Between Jan 2000 and Dec 2023, in Cochrane Reviews, Cochrane Protocols, Trials, Clinical Answers, Editorials, Special Collections.*

*Results: 81*
