# Supplementary material for: A Systematic Review Examining the Association of Falls With Diabetes‐Related Foot Ulcers
Source: J Foot Ankle Res. 2025 Jun 16;18(2):e70057. doi: 10.1002/jfa2.70057 (PMC12170942; doi:10.1002/jfa2.70057)
Supplement: Supplementary file 2 — Supporting Information S2 [file JFA2-18-e70057-s002.docx]

| Domain | Criteria | Points available |
| --- | --- | --- |
| Selection | Patients are representative of the average patient with diabetes in the community | 1 |
|  | Non-exposed cohort was drawn from the same community as the exposed cohort | 1 |
|  | Ascertainment of DFU was clinically measured or obtained through structured interview/exam | 1^α^ |
|  | History of DFU was well-defined in the control group   - 2 points for no history of DFU  - 1 point for prior DFU  - 0 points if undefined | 2^β^ |
| Comparability | Study controls for prior falls [Wettasinghe 2020] | 1 |
|  | Study controls for another important factor: age, insulin use, peripheral neuropathy | 1 |
| Outcome | Ascertainment of fall was via independent blind assessment or record linkage | 1 |
|  | Was there at least 6 months of follow-up to capture a fall? | 1 |
|  | Complete follow-up, or more than 90% follow-up with the number lost to follow-up accounted for | 1 |
| Total |  | 10 |

Supplementary Table . The modified version of the Newcastle-Ottawa Scale used in this systematic review to assess the risk of bias of observational studies is identical to the original but awards an extra point in the selection category based on the characteristics of the control group used in the study.

^α^DFU diagnoses that were ascertained from databases using ICD-9 or ICD-10 codes were awarded points.
^β^If DFD history was provided, but not specifically DFU, then the study was not awarded points.

Bias risk categories
7-10: Low risk of bias
4-6: High risk of bias
1-3: Very high risk of bias
