# Supplementary material for: A Systematic Review Examining the Association of Falls With Diabetes‐Related Foot Ulcers
Source: J Foot Ankle Res. 2025 Jun 16;18(2):e70057. doi: 10.1002/jfa2.70057 (PMC12170942; doi:10.1002/jfa2.70057)
Supplement: Supplementary file 4 — Supporting Information S4 [file JFA2-18-e70057-s001.docx]

| **Reference** | **Country** | **Study design** | **Setting** | **Cohort groups** | **Follow-up period** | **n** | **Age (mean ± SD)** | **Males** | **Diabetes duration** | **Insulin use** | **Falls history** | **CVD** | **CKD** | **PAD** | **Retinopathy** | **Neuropathy** | **BMI** | **Use of mobility device** |
| --- | --- | --- | --- | --- | --- | --- | --- | --- | --- | --- | --- | --- | --- | --- | --- | --- | --- | --- |
| Allen et al., 2017 [38] | USA | Retrospective cohort | Hospital | DFU | NR | 3586 | 63.5 ±10.6 | 3476 (96.9) | NR | NR | NR | 30 (0.8) | 19 (0.5) | 1415 (39.5) | 86 (2.4) | 833 (23.2) | NR | NR |
|  |  |  |  | No DFU |  | 40938 | 64.4 ±11.5 | 39197 (95.8) |  |  |  | 245 (0.6) | 111 (0.3) | 3784 (9.2) | 232 (0.6) | 2489 (6.1) |  |  |
| Bicer et al., 2020 [28] | Turkey | Cross-sectional | Hospital | DFU | 1 year | 83 | <65: 42 (50.6)  ≥65: 41 (49.4) | 42 (50.6) | NR | 69 (83.1) | NR | NR | NR | 69 (83.1) | NR | 64 (77.1) | Overweight: 28 (33.7) Obese: 33 (39.8) | 45 (54.2) |
|  |  |  |  | No DFU |  | 217 | <65: 133 (61.3) ≥65: 38 (38.7) | 82 (37.8) |  | 135 (62.2)  P=0.001 |  |  |  | 209 (96.3)  P=0.000 |  | 123 (56.7)  P=0.001 | Overweight: 73 (33.6) Obese: 84 (38.7) | 50 (23.0)  P=0.000 |
| Fang et al., 2023 [37] | USA | Retrospective cohort | Community | DFD^α^ | NR | 326 | 64.0 ± 5.2 | 179 (54.9) | <9 years: 134 (41.1) | 99 (30.4) | Never | 85 (26.1) | 100 (30.7) | 13 (4.0) | 89 (27.3) | NR | Overweight: 102 (31.3) Obese: 190 (58.3) | NR |
|  |  |  |  | No DFD^α^ |  | 1102 | 63.2 ± 5.7 | 579 (52.5) | <9 years: 612 (55.5) | 201 (18.2) | Never | 226 (20.5) | 258 (23.4) | 56 (5.1) | 155 (14.1) |  | Overweight: 381 (34.6) Obese: 609 (55.3) |  |
| Seo et al., 2023 [36]^γ^ | Korea | Cross-sectional | Hospital | DFU^β^ | 1 year | 60 | 64.9 ± 10.7 | 50 (71.4) | ≤10 years: 33 (47.1) | NR | NR | NR | NR | 49 (70.0) | 33 (47.1) | 46 (65.7) | NR | 35 (50) |
|  |  |  |  | No DFU^β^ |  | 10 |  |  |  |  |  |  |  |  |  |  |  |  |
| Data are presented as n (%) unless stated otherwise. CVD = cardiovascular disease; CKD = chronic kidney disease; PAD = peripheral artery disease; NR = not reported. Seo presented their data in two groups, fallers and non-fallers, but both groups had DFD. ^α^DFD was defined as a composite of foot ulceration, cellulitis, gangrene, osteomyelitis or paronychia. ^β^DFD was defined as including open wounds, bleeding disorders and ulcers in the foot.  ^γ^Seo et al. did not report on the baseline characteristics of their DFU and no DFU groups separately. | | | | | | | | | | | | | | | | | | |

Appendix 4 – Full table of patient characteristics
